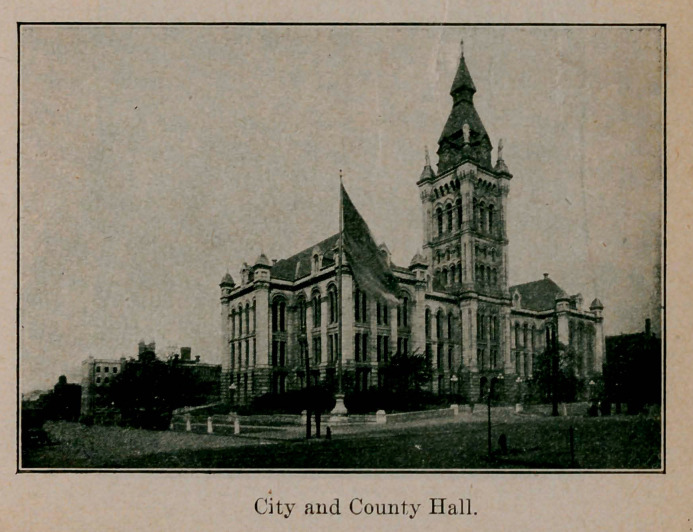# Topics of Public Interest

**Published:** 1915-04

**Authors:** 


### TOPICS OF PUBLIC INTEREST.

---

**Distribution of Wealth of U. S.** The federal income tax reports for the year 1914, showed that 44 persons had incomes of a million or over and that 357,598 had incomes of $3,000 or over. Estimating twenty million average families, about 1½ per cent. had incomes of more than $3,000 net. This is probably an underestimate.

---

**Courses Taken By Students in Arts and Sciences, U. of B.:**

| Subject | Instructor. |
|---|---|
| *Mathematics 1—4 hours a week | Mr. Sherk |
| *English—3 hours a week | **Mr. Goetz** |
| English Literature—1 hour a week | **Mr. Goetz** |
| *French 1—3 hours a week | Mr. Casassa |
| French 2—3 hours a week | Mr. Casassa |
| *German 1—3 hours a week | Mr. Oncken |
| German 2—3 hours a week | Mr. Oncken |
| Italian (omitted 1914-15) 3 hours a week | Mr. Oncken |
| History (modern European) 3 hours a week | **Mr. Park** |
| *Chemistry—6 hours a week | **Prof. Sy** and **Mr. Ralph** |
| *Biology—6 hours a week | **Mr. Shilliday** |
| *Physics—6 hours a week | Prof. Thomas and Prof. Piper |
| Mechanical Drawing—4 hours a week | Mr. Hopkins |
| Quantitative Analysis—6 hours a week | **Mr. Ralph** |
| Organic Chemistry—6 hours a week | **Prof. Sy** |
| Mathematics 2—5 hours a week | Mr. Sherk |

* First year subjects; others are taken by second year students and special students, as well as a few first-year.)

Those whose names are printed in black letters devote their entire time to instruction.

Organization of Faculty: Executive Committee—Messrs. Gotez, Park, Sherk, Thomas. Chairman of Faculty, Mr. Goetz. Secretary and Treasurer of Department—Mr. Park. One stenographer.

The studies for students entering the medical department in a year, are prescribed: **i. e.**, chemistry, physics, biology, English, and either French or German.

---

**Prisoners of War.** Germany reported that, on January 1, 1915, she held the following: French, 219,364, including 3,452 officers and 9 generals; Russian, 309,869, including 3,557 officers and 18 generals; Belgian, 37,464 including 609 officers and 9 generals; English, 19,316, including 422 officers. Total, 586,013.

---

**Uniform Food and Drug Legislation** is urged by a committee of the Chamber of Commerce of the U. S. A. consisting of Mr. Willoughby M. McCormick, chairman, McCormick & Company, Baltimore, Md.; Mr. A. J. Porter, Shredded Wheat Company,

Niagara Falls, N. Y.; Mr. B. L. Murray, Merck & Company, New York City, N. Y.; M. Theodore F. Whitmarsh, Francis H. Leggett & Company, New York City, N. Y.; and Mr. John A. Green, National Association of Retail Grocers, Cleveland, Ohio.

Charles Wesley Dunn, Counsel of the American Specialty Manufacturers Association calls attention to a number of inconsistencies; some of which are appended. Following the model of the Federal (Harrison) Narcotic Law, a proposed uniform state law is submitted.

In Colorado the sale of a food containing formaldehyde, and in North Dakota the sale of a food containing salicylic acid, is prohibited, while in Oregon a food containing formaldehyde, salicylic acid or other poisonous substances may be sold if the quantity or proportion thereof is indicated upon the label.

The seven certified coal-tar dyes may be used to color foods, generally, under the Federal and State laws, except in Minnesota and North Dakota, in these two states being condemned as unwholesome.

It is ruled under The Pennsylvania Pure Food Law that the seven certified coal-tar dyes may be used in all foods, as not injurious, while in the Fruit Syrup Law of the same State, the use of the same seven coal-tar dyes is expressly prohibited by statute as injurious to health.

The coloring of vinegar is prohibited in Connecticut and other states and permitted under The Federal and several other State Laws.

Vinegar must contain not less than 4 grams of acetic acid per one hundred cubic centimeters under the Federal and many State laws, and not less than 4½ per cent. by weight of absolute acetic acid in Massachusetts and South Dakota.

In Delaware, Nevada and Pennsylvania the statutory standard loaf of bread must weigh at least one pound, while in Kansas, Massachusetts and North Dakota the statutory standard for a loaf of bread is two pounds.

A barrel of potatoes to be legal in Connecticut must contain 172 pounds while in Tennessee a barrel of the same potatoes need only weigh 150 pounds.

Drugs named the United States Pharmacopoeia and National Formulary may vary from the official standard if the actual standard is plainly declared upon the label, under the Federal and many state laws, while under the laws of other states such drugs could not legally be sold.

A drug that is defined as a Poison in the law of one state is not defined as a Poison and need not be so sold in another state.

Toilet preparations may not contain wood alcohol in one state and may legally contain wood alcohol in another state.

The distribution of the samples of poisonous or dangerous drugs upon the doorstep and in the yards, etc., where children may obtain them, is prohibited in one state and unrestricted in another state.

---

**Smoking in Street Cars.** The Bulletin of the Buffalo Dept. of Health states that it has heard no good argument for smoking anywhere. It is a foolish and occasionally dangerous habit. Especially when indulged in its mildest form, the most estimable persons have declared that it leads to arson, theft, murder, insanity, sexual depravity and perversion. But, there is this to be said on the other side of the subject: granted that a man smokes at all, he is rather more apt to want to smoke at the hours that he uses the street cars than at others; smoking passes the time and tends to discourage reading which is harmful to vision in an oscillating vehicle; enough persons smoke so that a fair estimate of space can be made for them and this has proved feasible both on steam and electric roads. So far as the non-smoker is concerned, tobacco smoke is seldom harmful and, as a rule, not more offensive than onions, garlic, Limburger cheese and perspiration, all of which are freely admitted to public vehicles, and with no such segregation as the smoker willingly submits to and even prefers. And, it should be realized that we are just about at the limit of tolerance of intolerance in this country. What we most need is a new commandment "Thou shalt not say shalt not to thy neighbor."

---

**Massachusetts** is confronted, like New York and some other states with a conflict between state bills and the Harrison anti-narcotic law. The Boston M. & S. Journal discusses the matter editorially but, while offering the obvious suggestions as to the necessity of a uniform and reasonable law, is no more successful than this journal in advising the physician how to conform to the present unsettled state of affairs in a satisfactory manner.

---

**The Samuel D. Gross Prize of $1,500** has been awarded by the Philadelphia Academy of Surgery to Dr. John Lawrence Yates of Milwaukee for an essay on Surgery in the Treatment of Hodgkin's disease.

---

**Military Statistics.** Australia, with a total population of about 5 million, is thus divided for military purposes: 1—unmarried men, 18 to 35, 525,000; 2—unmarried men, 35 to 45, 87,250; 3—married, 18 to 35, 232,150; 4—married, 35 to 45, 232,250; 5—single and married, 45 to 60, 336,700. Total theoretic military strength, 1,414,200. It is estimated than 20 per cent. will be medically or otherwise "unfit." As a general rule, the full military strength of a nation may be estimated at one quarter of the population, the sexes being equally divided, and about half the population being above or below the 25 year line. Approximately, each year of life from 15 to 34 corresponds to 2 per cent. of the total population, this estimate being a fraction high. The population of Canada is about 7½ millions, the estimates corresponding.

---

**The Mayo Foundation.** Drs. Wm. J. and Charles H. Mayo of Rochester, Minn., have offered to the University of Minnesota, a fund of one million dollars, to establish an institution for medical and surgical research, at Rochester, but under the auspices of the University, the course to be pursued by graduates and to lead to a special degree. The Faculty of the Medical Dept., by a vote of 39 to 26, have approved the offer.

---

**The Massachusetts Medical Society** published a list of 3,523 active and retired fellows, January 1, 1915. The total number of physicians listed by Polk for the state in 1914 was 5,346. As directory lists contain the names of many persons not in practice, it may safely be estimated that 75 per cent. of the profession of Massachusetts is organized.

---

**Belgian Relief Fund.** Over three million dollars have been disbursed for the relief of our profession in Belgium but the need is still great. Do not forget the American Medicine Fund.

---

**Absinthe has been Forbidden** in Italy, as well as France.

---

**6,500 of 14,000 French Army Surgeons** are at the front.

---

**The Harrison Anti-Narcotic Law.** In our last issue, we stated that this law apparently exempted physicians employing narcotics only in conservative doses and in good faith, in practice, and not acting as suppliers for patients addicted to the habit. Dr. C. F. Taylor of the Medical World communicates the following letter, with copies of correspondence substantiating the fact that the rulings of the Commissioner of Internal Revenue read into the law a nullification of the exemptions. We have elsewhere stated our opinions as to legislation by executive officers and courts. The constitution of the United States plainly expresses the paramount authority of the individual states in local matters and the United States has allowed the license of physicians to be a strictly local matter. The constitution has also expressed itself plainly as to retro-active legislation. Both the State, by the Boylan law and the nation by the Harrison law, have curtailed the privileges of physicians already secured by license, in conformity with existing laws, and the Harrison law is declared to apply to local, intra-state practice. Waiving the question as to whether these laws are unconstitutional by being retro-active, the medical profession is confronted with a peculiar situation. If the two laws were essentially contradictory, the problem would be easier. The individual physician, in that case, could obey one law or the other and leave it to the state and national authorities to fight it out. But, with the two laws in substantial harmony but differing in detail, each providing for certain formalities in the way of blanks, registration, stock and note-taking, etc., how shall one proceed? We expect, as soon as possible to present expert legal opinions from Hon. Henry W. Hill and perhaps others. Dr. Taylor's letter is appended:

Dear Doctor:—These are the words of the new National Narcotic Law:

"Nothing contained in this section shall apply—(a) To the dispensing or distribution of any of the aforesaid drugs to a patient by a physician, dentist or veterinary surgeon registered under this act **in the course of his professional practice only**; provided, that such physician, dentist or veterinary surgeon shall keep a record of all such drugs dispensed, showing the amount dispensed or distributed, the date and the name and address of the patient to whom such drugs are dispensed or distributed, except such as may be dispensed or distributed to a patient upon whom such physician, dentist or veterinary surgeon shall **personally attend**; and such record shall be kept for a period of two years from the date of dispensing or distributing such drugs, subject to inspection, as provided in this act."

See particularly the words which I have underlined. The following is the ruling of the Bureau of Internal Revenue:

"Where a physician personally visits a patient **and administers** any of the drugs coming within the scope of the Harrison Act, he is not required to keep a record of such administration, but where he leaves a supply of any of these drugs or preparations to be taken by the patient in the physician's absence, he will be required to keep a record of such drug or preparation, the same as he would in his office. A physician must keep a record of all drugs or preparations dispensed or distributed in his office, whether administered personally or given to the patient to be carried away with him."

I consider this ruling very unjust to physicians. In this connection the accompanying correspondence with the Bureau of Internal Revenue may interest you. I hold that "personally attend in the course of professional practice" should include the leaving of necessary medicines for use between visits; and also include the dispensing of needed medicines at the office after consultation; that the words of the law do not mean "personally administer."

Very sincerely yours,
C. F. TAYLOR.

---

**A Widowed Mothers' Pension Bill** has been introduced into the New York Senate by William H. Hill. Local boards are provided for counties, except New York City which has an analogous provision. The members are appointed by judges or the Mayor, respectively, and serve without pay, the county superintendent of poor or commissioner of public charity being an ex-officio member. The local boards are made more or less subject to the state board of charities and, so far as appropriations and limitations of apportionments are concerned, to the county supervisors or corresponding boards for New York City. The local boards pass upon the suitability of the mother to bring up her children, the necessities of the individual case and the amount of allowance.

---

**Opposition to Bond Issue for Tuberculosis Hospital for Buffalo.** The Aldermanic Finance Committee strongly disapproves of the bond issue for $600,000 on the grounds that the present municipal hospital and county hospital care for 202 and 94 cases respectively, and that the proposed hospital would provide for only 260 cases. Considering that the total estimate of cases is 5,000 to 7,000, they consider the gain imperceptible. They also suggest wiping out the present (two) special hospital commissions as well as the anti-tuberculosis society and centralizing the management.

---

**A Bill to Require Everyone Seeking Admission to a Hospital or Sanitarium,** to lay before the Supt. of the Poor, his family history and resources, has been introduced by Senator George F. Thompson of Niagara County. The State Charities Aid Association states that this bill has been introduced in essentially the same form for four years and combats it strongly on the ground that it is being supported by some power behind the various introducers for unworthy motives; also that "a sick man is merely a patient and not a pauper," that, "for the protection of the community the sick should be cared for and a patient should not be subjected to the indignity of applying to the Supt. of the Poor for a certification that he has or has not sufficient funds."

Note—If the bill were in the opposite form, we could easily see how a disposition to shirk personal responsibility for dependents upon the state could constitute an interested motive or how a distribution of patronage could be turned to political ends. There may be objectionable features in this bill but sick relief is a time-honored form of charity and it is sound sense to require a rigid examination of applicants for any form of charity. So far as our experience goes, no honest applicant for public or private philanthropy of any kind has ever objected to stating his reasons to and having them verified by any proper agent. We certainly cannot condemn this bill on the general principles stated. If there are special features which deserve condemnation, they should be clearly pointed out.

---

**U. S. Civil Service Examination for Mine Surgeon.** A vacancy exists in the Bureau of Mines at Pittsburgh, at a salary of $2,400 to $2,700. Eligibles may also be appointed to other positions as they occur. At least two years' experience with industrial workers is required. Applicants should secure Forms 304 and 2095 from the U. S. Civil Service Commission, Washington, at once, as credentials must be filed by April 20. No formal examination will be held. Rating will be made as follows: Education 30 weights, Experience 50, Publication or thesis 20.

---

**Vaccination Bills.** The Tallett bill, requiring vaccination in third-class cities and rural communities only in time of epidemic, passed the Assembly March 4, by a vote of 81 to 28. The Senate Committee had a hearing on the Spring Bill repealing the present compulsory vaccination law, in which the discussion bore largely on the unfairness of discriminating between public and private schools. Dr. Linsley S. Williams of the State Health Department spoke in favor of this bill. This Journal stands unequivocally in favor of vaccination all the time, as a matter of medical opinion. But in view of public opinion, we are inclined to believe that some relaxation of the present law may ultimately be beneficial. If vaccination—with reasonable allowance for revaccination—does not protect the vaccinated, as most medical men believe, it is time that we knew it. We have all confidence that it does. Hence, the effect of relaxation of the present law would be about as follows: A period of several years in which, by the persistence of the immunity at present nearly universal, there will be no untoward result from the yielding to the demands of the antis, who will accordingly say "I told you so." Then, suddenly, small pox will develop in a community, with a pretty sharp distinction among severe cases in the unvaccinated; mild, varioloid cases in part of those vaccinated in childhood and with revaccination neglected; immunity among those recently vaccinated. For the reasons stated, there will be only a few cases of the disease and, by the law of chance, about one in ten of these will die. And, with this demonstration conspicuously before the people, there will be no trouble in securing radical vaccination laws that will endure for a generation or more. Simply as a matter of logical fairness, a further alternative must be admitted; that the views of the majority of the medical profession, apparently supported by the best of evidence in the past, are all wrong. And such a demonstration, though scarcely conceivable, would be of equal value for the medical profession upholds vaccination as a matter of scientific belief, not as a fetich.

---

**Hydrastis.** Till 1880, the value was about 10 cents a pound. In 1911, it was listed at $3 and has risen to about $5 with a sharp tendency to further increase. It is estimated by the Farmers' Bulletin that it costs $1,500 to start an acre of the crop, that there will be no return for 4 or 5 years and that, thereafter, a yield of about 2,000 pounds a year can be expected. Meantime, there is a large amount of this valuable drug in waste land, awaiting any school boy who will take the trouble to learn how to identify it, and how to gather it. We call the attention of philanthropists interested in giving children vacations and means of earning money to this field.

---

**Nurses' Registration Bill.** Assemblyman Tallett at the request of the State Department of Education, has introduced a bill providing for the distinction of graduate and practical nurses, for regulating the practice of nursing and placing it under the control of the Regents.

---

**Foot and Mouth Disease** has developed in a man employed on a farm near Utica.

---

**The Buffalo General Hospital Suffered a Loss of $5,000** from a fire in the laundry, February 26.

---

**Excursion to California.** The National Laboratories of Pittsburgh, have (or, according to the elastic syntax of English, has) organized an excursion starting from Pittsburgh, Monday June 14, and including the A. M. A. meeting June 21 to 25. The entire trip will occupy about three weeks. The entire expense will be $137, including sleeper fares, full meals on trains, use of sleepers at all stopping points, etc. Kansas City, Colorado Springs—with side trips to Pike's Peak, Garden of the Gods, etc.—Grand Canyon, Glenwood Springs, Salt Lake City, and various side trips in California, will be included.

---

**Early Marriage Question.** The Council of the American Genetic Association has secured an extension of the time limit of the prize offered by Caspar L. Redfield to December 31, 1915. $200 is offered for evidence in favor of early marriage, the object of the Association being to secure repeal of laws allowing marriage at such ages as 15 for males (as in three states) and 13 for females (as in four states). For further information, address C. L. Redfield, 525 Monadnock Block, Chicago.

---

**The Rudolph Virchow Medal** has been awarded to Dr. Karl Poldt, Emeritus Professor of Anatomy, University of Vienna.

---

**Tetanus.** The experience of the present war, as contrasted with that of the Boer war, brings up the question as to whether tetanus and certain forms of gas infection, etc., are strictly "soil diseases" or whether they are more appropriately to be regarded as manure diseases and whether the specific habitual host, as the horse for tetanus, may be further distinguished. While there is a tendency to regard manure as the ultimate

City and County Hall.

carrier, we believe that no thorough study of this phase of etiology has been made and the time is ripe for communications on the subject.

## Figures and Tables

**Figure f1:**